# Draft Genome Sequences of *Fusobacterium nucleatum* ChDC F145, ChDC F174, ChDC F206, and ChDC F300, Isolated from Human Subgingival Plaques in the Republic of Korea

**DOI:** 10.1128/genomeA.01233-13

**Published:** 2014-01-23

**Authors:** Soon-Nang Park, Eugene Cho, Yun Kyong Lim, Hwa-Sook Kim, Dae-Soo Kim, Jaeeun Jung, Jeong-Hun Baek, Eojin Jo, Young-Hyo Chang, Jeong Hwan Shin, Sang-Haeng Choi, Jihee Kang, YongUn Choi, Hong-Seog Park, Hongik Kim, Joong-Ki Kook

**Affiliations:** a Korean Collection for Oral Microbiology and Department of Oral Biochemistry, School of Dentistry, Chosun University, Gwangju, Republic of Korea; b Department of Dental Hygiene, Chunnam Techno University, Chunnam, Republic of Korea; c Human Derived Material Center, Korea Research Institute of Bioscience and Biotechnology (KRIBB), Daejeon, Republic of Korea; d Macrogen, Inc., Gasan-dong, Seoul, Republic of Korea; e Korean Collection for Type Cultures, Biological Resource Center, KRIBB, Daejeon, Republic of Korea; f Department of Laboratory Medicine, Busan Paik Hospital, Inje University College of Medicine, Busan, Republic of Korea; g Research Institute of Unitech Science Co., Ltd., Daejeon, Republic of Korea; h R&D Division, Vitabio, Inc., Daejeon, Republic of Korea

## Abstract

Recently, five strains were isolated from human subgingival plaques and were proposed as a novel subspecies of *Fusobacterium nucleatum*. Here, we report the draft genome sequences of the strains, except one for which the draft sequence was already introduced.

## GENOME ANNOUNCEMENT

*Fusobacterium nucleatum* might play an important role in the initiation and progression of periodontal diseases (1, 2). *F. nucleatum* was classified as five subspecies, i.e., *nucleatum*, *polymorphum*, *vincentii*, *animalis*, and *fusiforme* (3, 4), and subspecies *vincentii* and *fusiforme* were reclassified as a single subspecies by phylogenetic analysis using a single sequence (24,715 bp) that concatenated 22 housekeeping genes (5). Recently, five strains of *F. nucleatum*, ChDC F128 (KCOM 1249), ChDC F145 (KCOM 1253), ChDC F174 (KCOM 1256), ChDC F206 (KCOM 1258), and ChDC F300 (KCOM 1268), were isolated from human subgingival plaques in the Republic of Korea, and proposed as a novel subspecies (6).

Draft sequencing was performed by the Macrogen Co. (Seoul, Republic of Korea) using the Illumina Hiseq 2000 system sequencing technology. We constructed 101 paired-end sequencing libraries with insert sizes of about 200 bp and generated 49,219,612 bp, 55,803,856 bp, 51,102,948 bp, and 47,784,704 bp of usable sequences from ChDC F145, ChDC F174, ChDC F206, and ChDC F300, respectively. We assembled the reads using SOAPdenovo (http://soap.genomics.org.cn). SOAPdenovo v. 1.05 was run with option K79 and the configuration options reverse_seq of 0 (standard mate-pair orientation), asm_flags of 3 (try harder to build large contigs), and rank of 1 (reads were used while scaffolding). The assembled reads were assembled into 164 contigs (with a size range of 200 to 152,765 bp), 118 contigs (211 to 165,391 bp), 173 contigs (205 to 234,406 bp), and 148 contigs (225 to 351,444 bp) from ChDC F145, ChDC F174, ChDC F206, and ChDC F300, respectively. The sizes of the draft genomes of ChDC F145, ChDC F174, ChDC F206, and ChDC F300 were 2,280,257 bp, 2,465,770 bp, 2,362,473 bp, and 2,441,149 bp, and the GC content percentages were 26.99%, 26.92%, 27.06%, and 26.95%, respectively.

Open reading frames (ORFs) were predicted and annotated using the Giimmer 3.02 modeling software package (7). The predicted protein sequences were annotated as gene ontology (GO) by the basic local alignment search tool (BLAST). Then, the GO classes were grouped into a total of 124 GO-Slim terms using the web tool CateGOrizer (8).

These draft genomes contained 2,128 protein-coding genes, 4 16S rRNA, 1 5S rRNA, and 32 tRNA genes (ChDC 145); 2,294 protein-coding genes, 2 23S rRNA, 1 16S rRNA, and 27 tRNA genes (ChDC 174); 2,282 protein-coding genes, 1 5S rRNA, and 29 tRNA genes (ChDC F206); and 2,318 protein-coding genes, 1 16S rRNA, 2 5S rRNA, and 27 tRNA genes (ChDC F300).

These genome sequences contained several key pathways for amino acids, carbohydrates, lipids, and organic acids. All the strains had the amino acid biosynthetic pathways for at least 10 amino acids: glutamate, glutamine, aspartate, asparagine, threonine, methionine, isoleucine, leucine, valine, and cysteine (from serine). The strains might biosynthesize three more amino acids (isoleucine, leucine, and valine) than the proposed type strain (ChDC F128) of the novel *F. nucleatum* subspecies (9). These genome sequences also contain the same virulence factors, such as butyrate fermentation-related genes, hemolysin, 5-nitroimidazole antibiotic resistance proteins, beta-lactamase, multidrug resistance proteins, and macrolide-efflux protein.

### Nucleotide sequence accession numbers.

These whole-genome shotgun projects have been deposited at DDBJ/EMBL/GenBank under the accession numbers ATKE00000000, ATKF00000000, ATKH00000000, and ATKG00000000 for ChDC F145, ChDC F174, ChDC F206, and ChDC F300, respectively. The versions described in this paper are the first versions, ATKE01000000 (ChDC F145), ATKF01000000 (ChDC F174), ATKH01000000 (ChDC F206), and ATKG01000000 (ChDC F300).
